# Gangrenous or Diphtheritic Angina or Croup

**Published:** 1872-11

**Authors:** 


Gangrenous or Diptheritic Angina, or Croup.—M. M. Bouchut and Labadie Lagrade read before the Paris Academy of Sciences a report touching the anatomical lesions occurring in the above mentioned diseases—(*Gaz. Hebdom.*)—in which the following points were set forth, viz: There is a primary lesion, found in ulceration of mucous membrane, or pseudo membrane, and secondary, cardiac or embolic. The latter account for the frequency of sudden and oftentimes unexpected death. Fibrinous deposits occur in the heart, and may be carried in the vessels to the lungs or brain, producing embolism, with its well-known symptoms, and mortal consequences.

